# Visual presentation of age differences in relative survival of hematological neoplasms in Sweden and the neighboring countries

**DOI:** 10.1007/s00277-025-06291-4

**Published:** 2025-03-06

**Authors:** Kari Hemminki, Frantisek Zitricky

**Affiliations:** 1 Biomedical Center, Faculty of Medicine and Biomedical Center in Pilsen, Charles University in Prague, Pilsen, 30605 Czech Republic; 2 Division of Cancer Epidemiology, German Cancer Research Center (DKFZ), Im Neuenheimer Feld 580, Heidelberg, 69120 Germany

**Keywords:** Prognosis, Periodic survival, Treatment, Cancer registry

## Abstract

For many hematological malignancies (HMs) survival among older patients is compromised. We want to test the most up-to-date age-group-specific survival differences in five hematological malignancies, Hodgkin lymphoma (HL), multiple myeloma (MM), chronic lymphocytic leukemia (CLL), acute myeloid leukemia (AML) and myeloproliferative diseases (MPD) in Sweden (SE) and compared these to Denmark, Finland and Norway. For analysis we apply a recently published metric for comparing and visualizing age-group-specific relative survival differences using data from the NORDCAN database between 1972 and 2021. Periodic changes in age-related deviation in SE survival showed increasing differences for AML and MM while for the other HMs the differences declined in the course of time. Country-specific differences were observed, for Finnish male CLL and female MPD deviations were larger than those for the other countries, both of which were explained by the deviant survival of the oldest patients. Age-related deviations in 5-year survival increased for AML and MM for which survival improvements have been achieved through intense treatment regimens but these are not offered to old patients because of risk of complications. Paradoxically, improving overall survival in AML and MM has contributed to the widening of the age gaps. For the remaining HMs, age-related deviations declined with time as even old patients benefitted from the survival improvements; most notably female MPD and CLL patients had hardly any age gaps. Age disparities are an issue in hematological malignancies, and an intense search for novel treatments also includes old patients with an example of success as a novel drug venetoclax.

**Supplementary Information:**

The online version contains supplementary material available at 10.1007/s00277-025-06291-4.

## Introduction

Overall survival has developed well in hematological malignancies (HMs) and 5-year relative survival in Sweden and the Nordic countries has reached 90% for Hodgkin lymphoma (HL), myeloproliferative diseases (MPD) and chronic lymphocytic leukemia (CLL) [1, 2]. Survival in multiple myeloma (MM) has also developed well to 60% but, among the main entities, survival in acute myeloid leukemia (AML) has remained the lowest (30%) [1–3]. From the 1960s onwards chemotherapies with cytotoxic alkylating agents such as nitrogen mustard, cyclophosphamide, chlorambucil and melphalan were introduced and improved first survival particularly in HL and acute lymphoblastic leukemia [4]. At around year 2000 the clinical use of the first successful targeted therapy started with a tyrosine kinase inhibitor imatinib in Philadelphia chromosome-related chronic myeloid leukemia coinciding with the introduction of monoclonal antibodies (rituximab) for B-cell diseases [2]. From then on molecular understanding of individual HMs led to numerous disease-specific targeted therapies and later immunotherapies [2, 5–7]. The only main therapeutic modality that has remained limited to HMs is hematopoietic stem cell transplantation (HSCT) important in MM and leukemias [4, 8–11].

Diagnostic age is an important determinant of survival in most adult cancers but its influence varies between cancers. Diagnostic age was considered in European survival studies of all main types of HMs diagnosed between years 2000 and 2007 [12]. The study concluded that age was a strong prognostic factors even when adjusting for comorbidities. A similar European study covering overlapping years of 1997 to 2008 found that the overall survival was increasing in most HMs but ‘only slightly’ in patients diagnosed at age 75 + years [13]. Age differences in survival in HMs have also been reported from the Surveillance, Epidemiology, and End Results (SEER) database [3, 14, 15]. Earlier Swedish and Danish studies have considered age-group specific survival differences in MM, AML and CLL [16–19]. We have recently analyzed age-specific survival in the Nordic countries in MM and AML and the diagnostic age is a strong prognostic factor for both [20, 21]. The available data on survival disadvantage in HMs among old patients have been the motivation to propose comprehensive approaches to therapy of old HM patients [22].

We apply here a novel metric for comparing and visualizing age-group-specific relative survival differences in common HMs of HL, MM, CLL, AML and MPD for a 50-year period. We show most data for Sweden (SE), the largest Nordic country, but for consistence some data are shown for Denmark (DK), Finland (FI) and Norway (NO). The selection of the HMs for the study was based on the availability of uniform, long-term survival data from the NORDCAN database (https://nordcan.iarc.fr/en). Non-Hodgkin lymphoma and chronic myeloid leukemia were excluded because of heterogeneity of disease types and acute lymphoblastic leukemia because of the variable age of onset that could not be controlled in the database. National survival trends for HM spanning a half century probably exist nowhere else outside the Nordic countries.

## Materials and methods

The NORDCAN database 2.0 (version 9.3) was accessed at the IARC website (https://nordcan.iarc.fr/en) in summer of 2024 [23–25]. Most analyses were based on Swedish data, except that for international comparison also Danish, Finnish and Norwegian data were used. The available data are grouped and not based on individual patients. We extracted data on case numbers and 1- and 5-year relative age-specific survival with 95%CIs for consecutive 5-year periods from 1972 until the end of 2021. NORDCAN uses the International Classification of Diseases (ICD) version 10 codes for HL (C81), MM (C90) and CLL (C91.1). Codes for AML were C92.0 (acute myeloid leukemia), C93.0 (acute monoblastic/monocytic leukemia), C94.0 (acute erythroid leukemia), C94.2 (acute megakaryoblastic leukemia), C94.4–5 (acute panmyelosis with myelofibrosis). Those for MPD were D45 (polycythaemia vera), D47.1 (chronic myeloproliferative disease), D47.3-D47.5 (essential hemorrhagic thrombocythemia, osteomyelofibrosis, chronic eosinophilic leukemia).

Relative survival in NORDCAN is calculated using the Pohar Perme method [26]. National general population life-tables stratified by sex, year and age were used in the calculation of expected survival. The ‘cohort approach’ was used to calculate relative survival in all but the last period, for which ‘period approach’ was used. Patients with only death certificate data and those 90 years or older were excluded. An inclusion criterion was a minimum 30 patients alive within given age group at start.

Age-specific 5-year relative survival for 10-year periods was calculated as an average of relative survival estimates available at NORDCAN for subsequent 5-year periods for each age-group.

The framework used to analyze dispersion of age-specific survival figures was described in detail previously [27]. To measure overall dispersion of age-specific survival, we calculated mean absolute deviation corresponding to average absolute deviation of individual age-specific estimates from their mean. A large deviation indicates presence of substantial differences in survival, pointing to survival disparities across the age spectrum. The 95% CIs of the mean absolute deviation estimates were derived from CIs provided by NORDCAN for relative survival data. The available asymmetric CIs on the survival scale correspond to log transformation of cumulative excess hazard, variability of which is approximated by normal distribution, allowing to calculate variance on the cumulative hazard scale. We next performed 10 000 Monte Carlo simulations, where cumulative excess hazard was drawn at random from the normal distribution, with estimated mean and variance. These values were transformed to the relative survival scale and used to derive random distribution for the metric, which represents estimated metric variability based on the random variability in the original data. The 2.5th and 97.5th quantiles of this random distribution correspond to the bounds of the 95%CIs. The obtained 95% CIs thus reflect uncertainty of the mean absolute deviation estimate based on uncertainty of the original NORDCAN age-specific relative survival estimates, under assumption of independence of individual estimates. Since the CIs are derived independently for individual temporal periods, they do not reflect possible temporal correlation in survival estimates, which should be recognized when considering temporal trends.

Differences in the mean absolute deviation were called significant when the 95%CIs were non-overlapping.

## Results

**Fig. 1:**
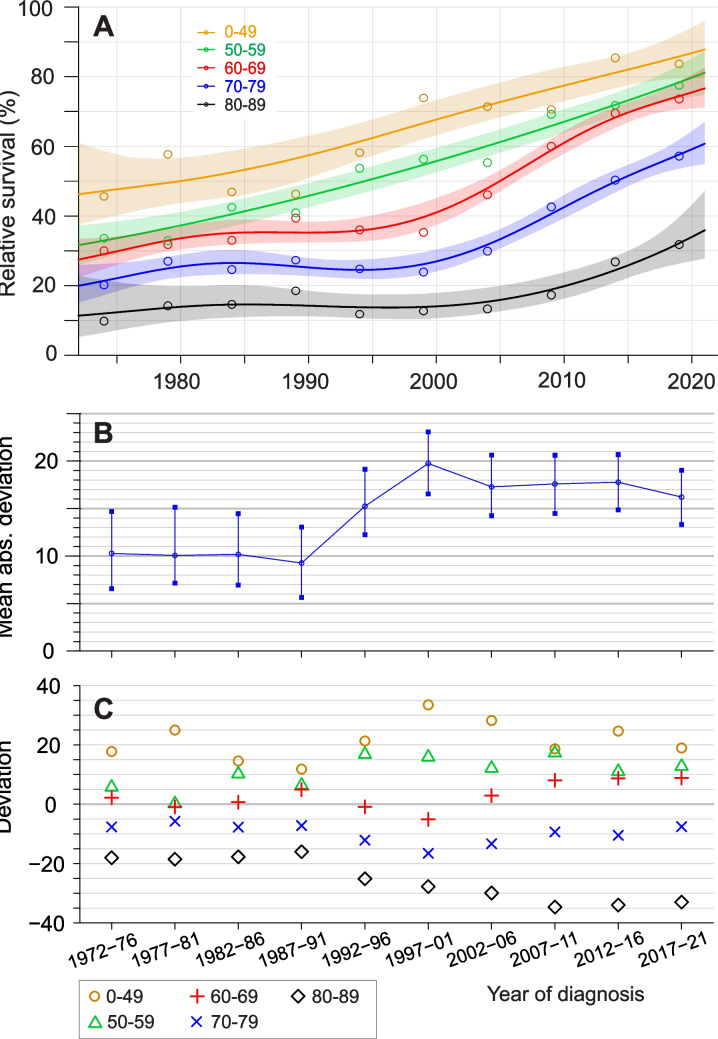
Application of the new metric to assess age-differences in relative survival. The example is on male 5-y relative survival in multiple myeloma in age-groups derived from NORDCAN and modelled to show 95% confidence intervals (95CIs) (**A**). Mean deviations are shown in (**B**) with 95CIs, and how these distribute between the defined age-groups is shown in (**C**)

We demonstrate the application of the novel metric with SE data on male MM 5-y survival in Fig. 1. Age-group-specific survival data (with 95%CIs) are shown in panel A. For the two youngest age-groups the survival graphs are linear and for older age-groups they are almost flat but bend upwards in step-wide later periods. Panel B considers the overall variation in survival levels between the age-group. The mean absolute deviation remained at 10% the first 20 years, then between 1990 and 2000 it climbed to a new level of 20% and then slowly declined again. At the time of the large increase in deviation many new treatments were introduced to relatively young patients which improved survival in their favor. This is actually demonstrated in panel C. The age gap widened between 1990 and 2000, and thereafter narrowed again between all age-groups but the oldest (80–89 year old).

The NORDCAN database was used for the period 1972 to 2021. We show age-standardized 5-year relative survival trends in men and women for SE (Supplementary Fig. 1). The figure distinguishes a group of HMs (MPD, CLL, HL) of a very good survival development, reaching now about 90%; the intermediary one of MM with survival of 65%; AML constitutes the only HM with survival below 40%, although 50 years ago the starting level was close to zero.

**Fig. 2:**
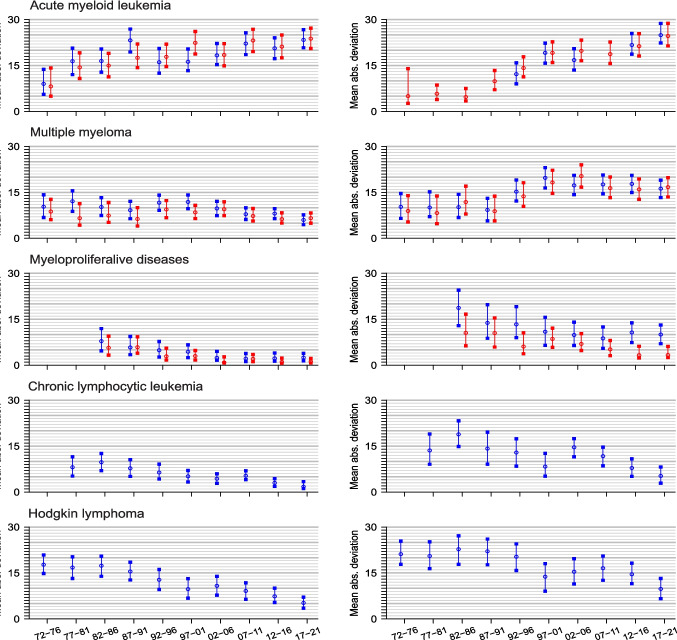
Age-related mean absolute deviations in 1- and 5-y relative survival in the HM covered, AML, MM, MPD, CLL, HL, in 5-y intervals, data points for men on the right and for women on the left

Age-related mean absolute deviations are shown in 1- and 5-y relative survival in the main HMs for which we have data in Fig. 2. For AML deviations for both 1- and 5-y survival increased periodically and reached the highest level, 25% for 5-year survival and only slightly less for 1-y survival. For MM deviation for 1-y survival declined from 10% while for 5-y survival it increased from 10 to 20% but turned down after 2010. For the other HMs deviations for 1-y survival were lower than those for 5-y survival, and for MPD and CLL hardly any deviation was observed for 1-y survival. For MPD deviations for female 5-y survival were much lower than male deviations.

**Fig. 3:**
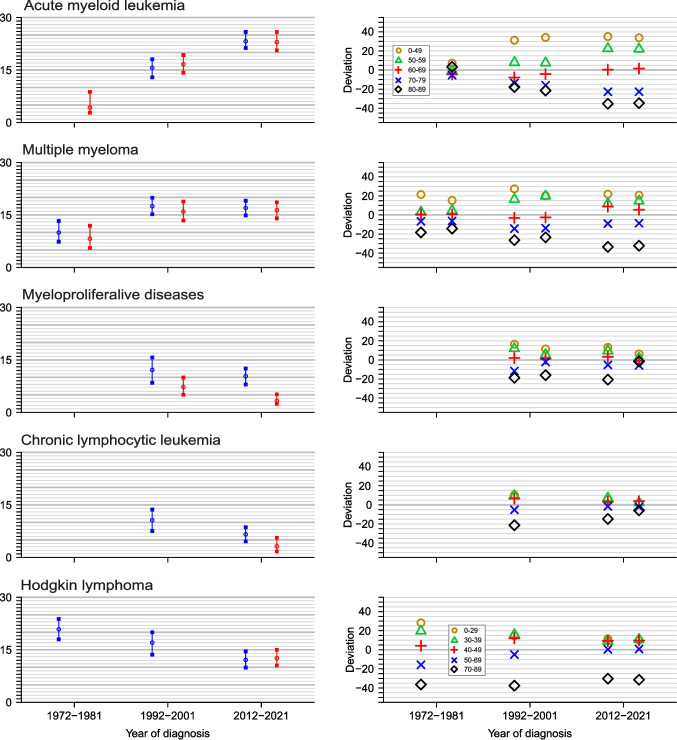
Age-related mean absolute deviations for 5-y relative survival in AML, MM, MPD, CLL, HL in three periods. When data are not shown, some data points were missing in the original NORDCAN data

Periodic changes in age deviation with data on individual age groups are shown in Fig. 3. In AML the time-dependent increase in mean deviation was due to the increasing survival gaps which in this case was mainly caused by improving survival in young patients. The situation was similar for MM. Deviations for MPD and CLL decreased with time and in 2012–21 female patients for both experienced no age differences. Age deviations decreased for HL and disappeared for patients younger than 70 years.

**Fig. 4:**
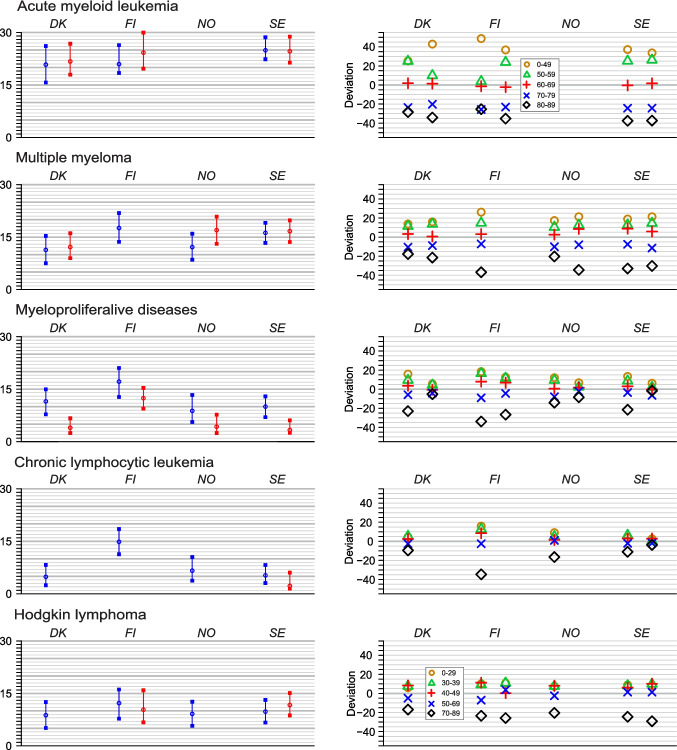
Age-related mean absolute deviations in 5-y relative survival in AML, MM, MPD, CLL, HL in the four countries, for men on the right and for women on the left

Comparison between the Nordic countries is shown in Fig. 4. Two country-specific differences were observed, one for CLL for which FI male deviation was larger (15%) than that for the other countries (< 7%); the other was for MPD for which FI females had larger deviation than their Nordic counterparts. Both were explained by the deviant survival of the oldest FI patients. For MPD a sex-specific difference was observed as the female deviations in DK and SE was lower compared to the male deviations; this was explained by very small age-group-specific survival differences in women.

## Discussion

Clinical oncology aims to provide best possible survival conditions for all patients but the reality has been and is that diagnostic age is an interfering factor for survival. For the present HMs with sufficient amount of age-specific data, overall survival differed vastly from those with excellent survival (MPD, CLL, HL) to MM with an intermediary survival and to AML low survival. In spite of these survival differences, the 50-year survival period shown for SE men and women in Supplementary Fig. 1 could document a survival increase of about 30% units for all of them.

Published survival literature has tried to capture the effect of age in different ways. The simplest option has been to carry out analysis in two or more age-groups and compared these to each other or to the largest age-group [28–31]. The problem in this approach is, as illustrated in the present study, that age distribution is cancer-dependent and may vary between populations. Another common approach is to present age-group specific survival with CIs (our Fig. 1A) or by visual comparison [12, 13, 26, 32, 33, 34, 35, 36]. The generated plots are illustrative but the approach has basically the same problems as the first one; moreover, as the age-groups are not equally large, it would be helpful to include an overall survival curve. The present approach summarizes the mean age distribution of the population and the deviation for the selected age-groups at the selected time points. Thus the present method is able to condense age-specific data into a tangible form. However it is non-informative of the actual survival and could be complemented by such data, as e.g., the set-up in Fig. 1. The survival method, whether crude, relative or net survival is not critical for the application [26].

In Fig. 2 we compared 1- and 5-y survival among SE patients and noted a high and increasing age-related deviation for AML (from 10 to over 20 % units). Early mortality is likely to be related to a fatal disease (such as AML), caused by infections, comorbidities and therapy resistance. For HL the initial deviation was close to 20% but it decreased to 5%. The decline is likely to be related to declining 1-y mortality affecting preferentially old patients. Early (1-year) mortality in HL has been due to heart and lung problems and infections which are seen even in the current first-line treatment with doxorubicin, bleomycin, vinblastine, and dacarbazine (ABVD); bleomycin in this cocktail is known to induce interstitial lung disease, otherwise a rare disease [37]. ABVD therapy displaced the earlier MOPP therapy (nitrogen mustard, vincristine, procarbazide and prednisone) because its toxicity; the change to a less toxic therapy may have contributed to the declining age deviation in HL.

Age-related mean absolute deviations increased in the course of calendar periods for AML and MM which was at the cost of widening gaps between age groups, evident in Fig. 3. As survival even for the oldest age groups (80–89 y) in AML and MM slightly increased, the huge gap to patients diagnosed before age 50 was explained by the success of novel treatments for young patients [20, 21]. AML survival has improved through intensive chemotherapy applied in curative intent using cytosine arabinoside (ara-C) and anthracyclines [38]. HSCT is often combined for treatment of fit high-risk AML patients younger than 70 years [39, 40]. However, as AML is a disease of old individuals, the age/fitness limitation restricts elderly patients from curative treatment as illustrated in Fig. 3. In the same figure data for MM also showed widening of age differences at around 1990–2000. HSCT was introduced for MM at around 1990, and fitness and age limits (first < 65 years) were also applied [41]. High-dose melphalan treatment was taken to use also at around 1990 [41]. Drugs with novel mechanisms of action for MM were introduced after year 2000 and these included immunomodulatory agents (e.g. thalidomide) and proteasome inhibitors (e.g. bortezomib) [41].

For MPD and CLL age differences declined with time and in 2012–21 female patients showed no age-differences (at the resolution of Fig. 3). NORDCAN-based survival data showed female 5-y relative survival for MPD in 2015–19 of 93% compared to male survival of 86% [2]. With such a high survival there is not much space for age-related differences. The three main types of MPD are myelofibrosis, polycythemia vera, and essential thrombocythemia which manifest with thrombohemorrhagic complications which can be alleviated by aspirin and hydroxyurea therapy [2]. Female survival is also better for CLL than male survival and 5-y survival is over 90% [2]. Therapies for CLL have evolved since the introduction of rituximab after year 2000, and some 30% of patients require no treatment for their CLL [2].

The novel survival metric can be used in concise country comparisons (Fig. 4). The example could pinpoint two country-specific differences both showing large age deviations in FI compared to the Nordic counterparts and the reason for these was the deviant survival of the oldest FI patients.

In conclusion, age-related deviation in 1-y survival data declined over calendar time for all HMs except for AML. Deviations for 5-y survival increased for AML and MM for which survival improvements have been achieved through intense treatment regimens; these are however not offered to old patients because of risk of complications. Paradoxically, improving overall survival in AML and MM has contributed to the widening of the age gaps. For the remaining HMs, age-related deviations declined with time as even old patients benefitted from the survival improvements. Most notably female MPD and CLL patients had very small mean absolute deviations. Age disparities are an issue in HMs and an intense search of novel treatments even for the old patients is ongoing. For old, unfit and relapsed AML patients the BCL2-inhibitor venetoclax has been approved to be used in combination with a hypomethylating agents [20, 42]. Venetoclax is also used in MM harboring a common translocation (11;14) as well as in CLL [43–45].

## Supplementary Information

Below is the link to the electronic supplementary material.

ESM 1 Overall 5-year relative survival in Sweden for the covered hematological malignancies from 1972–76 to 2017–21 based on the NORDCAN data. Male curves are solid and female ones dotted lines. (PDF 132 KB)

## Data Availability

No datasets were generated or analysed during the current study.

## References

[1] Hemminki K, Hemminki J, Försti A, Sud A (2022) Survival trends in hematological malignancies in the Nordic countries through 50 years. Blood Cancer J 12(11):150 36336699 10.1038/s41408-022-00728-z PMC9637692

[2] Hemminki K, Hemminki J, Försti A, Sud A (2023) Survival in hematological malignancies in the Nordic countries through a half century with correlation to treatment. Leukemia 37(4):854–863 36828868 10.1038/s41375-023-01852-w PMC10079539

[3] Pulte D, Jansen L, Brenner H (2020) Changes in long term survival after diagnosis with common hematologic malignancies in the early 21st century. Blood Cancer J 10(5):56 32404891 10.1038/s41408-020-0323-4 PMC7221083

[4] DeVita VT Jr, Chu E (2008) A history of cancer chemotherapy. Cancer Res 68(21):8643–8653 18974103 10.1158/0008-5472.CAN-07-6611

[5] Crisci S, Amitrano F, Saggese M, Muto T, Sarno S, Mele S et al (2019) Overview of current targeted anti-cancer drugs for therapy in onco-hematology. Medicina (Kaunas) 55(8):414 31357735 10.3390/medicina55080414 PMC6723645

[6] Olejarz W, Basak G (2023) Emerging therapeutic targets and drug resistance mechanisms in immunotherapy of hematological malignancies. Cancers 15(24):5765 38136311 10.3390/cancers15245765 PMC10741639

[7] Tang L, Huang Z, Mei H, Hu Y (2023) Immunotherapy in hematologic malignancies: achievements, challenges and future prospects. Signal Transduct Target Ther 8(1):306 37591844 10.1038/s41392-023-01521-5 PMC10435569

[8] Evens AM, Hutchings M, Diehl V (2008) Treatment of Hodgkin lymphoma: the past, present, and future. Nat Clin Pract Oncol 5(9):543–556 18679394 10.1038/ncponc1186

[9] Pui CH, Nichols KE, Yang JJ (2019) Somatic and germline genomics in paediatric acute lymphoblastic leukaemia. Nat Rev Clin Oncol 16(4):227–240 30546053 10.1038/s41571-018-0136-6

[10] Yang B, Yu R, Cai L, Bin G, Chen H, Zhang H et al (2019) Haploidentical versus matched donor stem cell transplantation for patients with hematological malignancies: a systemic review and meta-analysis. Bone Marrow Transplant 54(1):99–122 29988061 10.1038/s41409-018-0239-9

[11] Khan M, Maker AV, Jain S (2021) The Evolution of Cancer Immunotherapy. Vaccines (Basel). 9(6):614 34200997 10.3390/vaccines9060614 PMC8227172

[12] De Angelis R, Minicozzi P, Sant M, Dal Maso L, Brewster DH, Osca-Gelis G et al (2015) Survival variations by country and age for lymphoid and myeloid malignancies in Europe 2000–2007: Results of EUROCARE-5 population-based study. Eur J Cancer 51(15):2254–2268 26421827 10.1016/j.ejca.2015.08.003

[13] Sant M, Minicozzi P, Mounier M, Anderson LA, Brenner H, Holleczek B et al (2014) Survival for haematological malignancies in Europe between 1997 and 2008 by region and age: results of EUROCARE-5, a population-based study. Lancet Oncol 15(9):931–942 25030467 10.1016/S1470-2045(14)70282-7

[14] Pulte D, Jansen L, Castro FA, Brenner H (2016) Changes in the survival of older patients with hematologic malignancies in the early 21st century. Cancer 122(13):2031–2040 27163715 10.1002/cncr.30003

[15] Shallis RM, Wang R, Davidoff A, Ma X, Podoltsev NA, Zeidan AM (2020) Epidemiology of the classical myeloproliferative neoplasms: The four corners of an expansive and complex map. Blood Rev 42:100706 32517877 10.1016/j.blre.2020.100706

[16] Blimark CH, Vangsted AJ, Klausen TW, Gregersen H, Szabo AG, Hermansen E et al (2022) Outcome data from >10 000 multiple myeloma patients in the Danish and Swedish national registries. Eur J Haematol 108(2):99–108 34514635 10.1111/ejh.13707

[17] Juliusson G, Hagberg O, Lazarevic VL, Ölander E, Antunovic P, Cammenga J et al (2019) Improved survival of men 50 to 75 years old with acute myeloid leukemia over a 20-year period. Blood 134(18):1558–1561 31515252 10.1182/blood.2019001728 PMC6839949

[18] Naur TMH, Jakobsen LH, Roug AS, El-Galaly TC, Marcher CW, Nørgaard JM et al (2021) Treatment intensity and survival trends among real-world elderly AML patients diagnosed in the period 2001–2016: a Danish nationwide cohort study. Leuk Lymphoma 62(8):2014–2017 33711911 10.1080/10428194.2021.1893315

[19] da Cunha-Bang C, Simonsen J, Rostgaard K, Geisler C, Hjalgrim H, Niemann CU (2016) Improved survival for patients diagnosed with chronic lymphocytic leukemia in the era of chemo-immunotherapy: a Danish population-based study of 10455 patients. Blood Cancer J 6(11):e499 27834937 10.1038/bcj.2016.105 PMC5148052

[20] Hemminki K, Zitricky F, Försti A, Kontro M, Gjertsen BT, Severinsen MT et al (2024) Age-specific survival in acute myeloid leukemia in the Nordic countries through a half century. Blood Cancer J 14(1):44 38480693 10.1038/s41408-024-01033-7 PMC10937905

[21] Hemminki K, Zitricky F, Försti A, Silvennoinen R, Vangsted A, Hansson M (2024) Large differencies in age-specific survival in multiple myeloma in the nordic countries. Blood Cancer J 14(1):43 38467614 10.1038/s41408-024-01026-6 PMC10928156

[22] Cordoba R, Eyre TA, Klepin HD, Wildes TM, Goede V (2021) A comprehensive approach to therapy of haematological malignancies in older patients. Lancet Haematol 8(11):e840–e852 34624238 10.1016/S2352-3026(21)00241-6

[23] Engholm G, Ferlay J, Christensen N, Bray F, Gjerstorff ML, Klint A et al (2010) NORDCAN–a Nordic tool for cancer information, planning, quality control and research. Acta Oncol 49(5):725–736 20491528 10.3109/02841861003782017

[24] Pukkala E, Engholm G, Hojsgaard Schmidt LK, Storm H, Khan S, Lambe M et al (2018) Nordic Cancer Registries - an overview of their procedures and data comparability. Acta Oncol 57:440–455 29226751 10.1080/0284186X.2017.1407039

[25] Larønningen SAG, Bray F, Engholm G, Ervik M, Guðmundsdóttir EM, Gulbrandsen J, Hansen HL, Hansen HM, Johannesen TB, Kristensen S, Kristiansen MF, Lam F, Laversanne M, Miettinen J, Mørch LS, Ólafsdóttir E, Pejicic S, Petterson D, Steig BÁ, Skog A, Tian H, Aagnes B, Storm HH (2023) NORDCAN: cancer incidence, mortality, prevalence and survival in the Nordic Countries, Version 9.3 (02.10.2023)

[26] Pohar Perme M, Estève J, Rachet B (2016) Analysing population-based cancer survival - settling the controversies. BMC Cancer 16(1):933 27912732 10.1186/s12885-016-2967-9 PMC5135814

[27] Zitricky F, Hemminki K (2024) A metric for comparison and visualization of age disparities in cancer survival. Cancer Epidemiol 91:102586 38762920 10.1016/j.canep.2024.102586

[28] Arnold M, Rutherford MJ, Bardot A, Ferlay J, Andersson TM, Myklebust T et al (2019) Progress in cancer survival, mortality, and incidence in seven high-income countries 1995–2014 (ICBP SURVMARK-2): a population-based study. Lancet Oncol 20(11):1493–1505 31521509 10.1016/S1470-2045(19)30456-5 PMC6838671

[29] Quaglia A, Tavilla A, Shack L, Brenner H, Janssen-Heijnen M, Allemani C et al (2009) The cancer survival gap between elderly and middle-aged patients in Europe is widening. Eur J Cancer 45:1006–1016 19121578 10.1016/j.ejca.2008.11.028

[30] Withrow DR, Nicholson BD, Morris EJA, Wong ML, Pilleron S (2023) Age-related differences in cancer relative survival in the United States: a SEER-18 analysis. Int J Cancer 152(11):2283–2291 36752633 10.1002/ijc.34463

[31] Schuurman MS, Lemmens V, Portielje JEA, van der Aa MA, Visser O, Dinmohamed AG (2024) The cancer burden in the oldest-old: increasing numbers and disparities-A nationwide study in the Netherlands, 1990 to 2019. Int J Cancer 154(2):261–272 37664984 10.1002/ijc.34705

[32] De Angelis R, Sant M, Coleman MP, Francisci S, Baili P, Pierannunzio D et al (2014) Cancer survival in Europe 1999–2007 by country and age: results of EUROCARE–5-a population-based study. Lancet Oncol 15(1):23–34 24314615 10.1016/S1470-2045(13)70546-1

[33] Pilleron S, Charvat H, Araghi M, Arnold M, Fidler-Benaoudia MM, Bardot A et al (2021) Age disparities in stage-specific colon cancer survival across seven countries: An International Cancer Benchmarking Partnership SURVMARK-2 population-based study. Int J Cancer 148(7):1575–1585 33006395 10.1002/ijc.33326

[34] Hemminki K, Försti A, Hemminki A (2021) Survival in colon and rectal cancers in Finland and Sweden through 50 years. BMJ Open Gastroenterol 8(1):e000644 34272211 10.1136/bmjgast-2021-000644 PMC8287611

[35] Hemminki K, Försti A, Hemminki A, Ljungberg B, Hemminki O (2021) Progress in survival in renal cell carcinoma through 50 years evaluated in Finland and Sweden. PLoS ONE 16(6):e0253236 34157049 10.1371/journal.pone.0253236 PMC8219161

[36] Hemminki K, Försti A, Hemminki A, Ljungberg B, Hemminki O (2022) Survival in bladder and upper urinary tract cancers in Finland and Sweden through 50 years. PLoS ONE 17(1):e0261124 34982793 10.1371/journal.pone.0261124 PMC8726478

[37] Dores GM, Curtis RE, Dalal NH, Linet MS, Morton LM (2020) Cause-specific mortality following initial chemotherapy in a population-based cohort of patients with classical Hodgkin Lymphoma, 2000–2016. J Clin Oncol 38(35):4149–4162 32946352 10.1200/JCO.20.00264 PMC7723686

[38] Döhner H, Wei AH, Appelbaum FR, Craddock C, DiNardo CD, Dombret H et al (2022) Diagnosis and management of AML in adults: 2022 recommendations from an international expert panel on behalf of the ELN. Blood 140(12):1345–1377 35797463 10.1182/blood.2022016867

[39] Fey M, Dreyling M (2009) Acute myeloblastic leukemia in adult patients: ESMO clinical recommendations for diagnosis, treatment and follow-up. Ann Oncol 20(Suppl 4):100–101 19454422 10.1093/annonc/mdp141

[40] Tallman MS, Wang ES, Altman JK, Appelbaum FR, Bhatt VR, Bixby D et al (2019) Acute myeloid leukemia, version 3.2019, NCCN clinical practice guidelines in oncology. J Natl Compr Cancer Netw: JNCCN 17(6):721–49 10.6004/jnccn.2019.0028 31200351

[41] Hemminki K, Försti A, Houlston R, Sud A (2021) Epidemiology, genetics and treatment of multiple myeloma and precursor diseases. Int J Cancer 149(12):1980–1996 34398972 10.1002/ijc.33762 PMC11497332

[42] Gangat N, Karrar O, Iftikhar M, McCullough K, Johnson IM, Abdelmagid M et al (2024) Venetoclax and hypomethylating agent combination therapy in newly diagnosed acute myeloid leukemia: Genotype signatures for response and survival among 301 consecutive patients. Am J Hematol 99:193–202 38071734 10.1002/ajh.27138

[43] Eichhorst B, Niemann CU, Kater AP, Fürstenau M, von Tresckow J, Zhang C et al (2023) First-line venetoclax combinations in chronic lymphocytic leukemia. N Engl J Med 388(19):1739–1754 37163621 10.1056/NEJMoa2213093

[44] Khan WJ, Ali M, Hashim S, Nawaz H, Hashim SN, Safi D et al (2023) Use of venetoclax in t(11;14) positive relapsed/refractory multiple myeloma: A systematic review. J Oncol Pharm Pract 30(3):10781552231219000 10.1177/10781552231218999 38113108

[45] Raab MS (2024) Venetoclax in myeloma: to B, or not to B. Blood 143(1):4–5 38175678 10.1182/blood.2023022535

