# Supplementary figures and images for: Visual presentation of age differences in relative survival of hematological neoplasms in Sweden and the neighboring countries
Source: Ann Hematol. 2025 Mar 6;104(3):1985–93. doi: 10.1007/s00277-025-06291-4 (PMC12031788; doi:10.1007/s00277-025-06291-4)

Supplementary Fig. 1

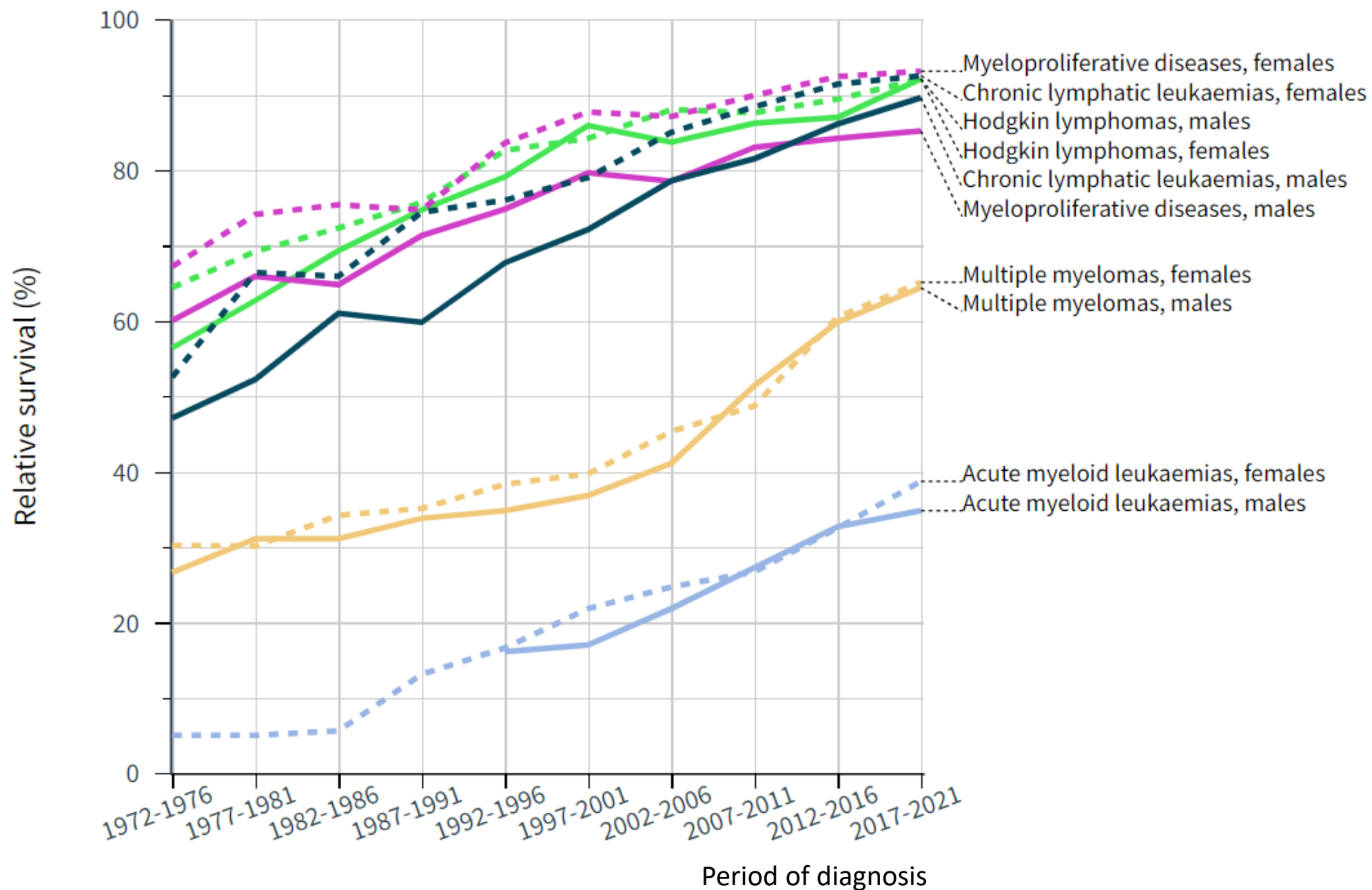

Supplement: Supplementary file 1 — Overall 5-year relative survival in Sweden for the covered hematological malignancies from 1972–76 to 2017–21 based on the NORDCAN data. Male curves are solid and female ones dotted lines. (PDF 132 KB) [file 277_2025_6291_MOESM1_ESM.pdf]
